# Molecular Regulatory Mechanisms of Mammary Gland Development: A Review

**DOI:** 10.3390/ani15233480

**Published:** 2025-12-02

**Authors:** Xiangnan Zhou, Abd Ullah, Limeng Shi, Manna Dou, Changfa Wang, Muhammad Zahoor Khan, Chunming Wang, Xinhao Zhang

**Affiliations:** College of Agriculture and Biology, Liaocheng University, Liaocheng 252000, Chinazahoorkhan@lcu.edu.cn (M.Z.K.)

**Keywords:** mammary gland development, hormonal regulation, Wnt signaling pathway, notch signaling, PI3K/Akt pathway, JAK-STAT signaling, lactation, involution

## Abstract

Mammary gland development represents a complex and highly regulated biological process that is critical for mammalian reproductive success. This review provides a comprehensive analysis of the molecular, cellular, and hormonal mechanisms governing mammary gland development across distinct developmental stages: embryonic, pubertal, pregnancy, lactation, and involution. Mammary organogenesis is orchestrated by multiple factors, including conserved genetic pathways, endocrine signaling cascades, and environmental stimuli. Drawing primarily from studies in rodents and dairy ruminants, recent advances in molecular biology have elucidated key signaling pathways, including Wnt, Notch, and TGF-β, which provide fundamental insights into mammary epithelial growth and differentiation. Furthermore, we examine the effects of external factors, including thermal stress, photoperiodic variation, and dry period duration on mammary function and lactational performance. The dry period, in particular, serves a critical role in tissue remodeling and preparation for subsequent lactation cycles. Through systematic review of physiological and molecular dynamics across developmental stages, this article identifies key genetic regulators and cellular mechanisms, offering valuable insights for optimizing milk production and composition in agricultural systems.

## Simple Summary

Mammary gland development is essential for lactation efficiency and animal productivity, yet the molecular mechanisms integrating hormonal regulation with environmental influences remain poorly unified across developmental stages. Integrating evidence from rodent models and dairy ruminants, this review provides a comparative synthesis of key signaling pathways, such as Wnt, Notch, PI3K/Akt, and JAK-STAT, governing mammary morphogenesis from embryogenesis through involution. It further highlights how nutrition, heat stress, photoperiod, and dry-period management affect these intrinsic molecular networks. By connecting fundamental biology with applied dairy management, this work offers a comprehensive framework for understanding mammary development and identifying strategies to improve milk production and gland health.

## 1. Introduction

The mammary gland is a compound tubuloalveolar organ with extensive branching morphology, representing a defining anatomical feature of the class Mammalia [1]. Mammary gland development constitutes a highly dynamic and precisely regulated biological process essential for mammalian reproductive fitness [1,2]. The development and physiological function of lactating mammary tissue are fundamental for providing essential nutrients to offspring during early postnatal development [1,3]. Mammogenesis proceeds through discrete developmental stages, each characterized by profound structural and functional remodeling regulated by complex interactions among hormonal, genetic, and environmental determinants [1,4]. Extensive research in this field has yielded significant insights into the molecular pathways and regulatory networks controlling these developmental processes and the sequential adaptation of mammary tissue to diverse physiological demands [1,5].

Throughout embryonic development, puberty, pregnancy, and lactation, the mammary gland undergoes extensive morphological and functional transformations [6,7]. These developmental transitions are orchestrated by a precisely coordinated interplay of hormones, growth factors, and genetic programming, ensuring optimal glandular capacity to support offspring at each developmental stage [1,8]. Early developmental events establish the architectural framework for subsequent milk synthesis, while pubertal development primes the gland for complete functional maturation during pregnancy and lactation [2,9]. Although these stages are temporally distinct, they collectively underscore the importance of elucidating the fundamental biological mechanisms governing mammary organogenesis [4,10].

Recent advances in molecular biology and genomics, particularly through studies in mice and dairy cattle, have significantly enhanced our understanding of the molecular and cellular mechanisms underlying mammary gland development [10,11]. Stage-specific gene expression programs and the activation of evolutionarily conserved signaling pathways are now recognized as critical regulators of mammary epithelial growth, differentiation, and functional specialization [1,11]. These insights have not only deepened our fundamental knowledge of mammary biology but have also revealed important implications for understanding pathological conditions, particularly breast cancer, which arises from disruptions in normal mammary gland function [10,12].

This article provides an in-depth discussion of the molecular alterations associated with various developmental stages of the bovine mammary gland. Furthermore, regulatory molecules controlling the transitions from one developmental stage to the next have been highlighted. Additionally, significant morphological changes during mammary gland development are thoroughly examined. Throughout the review, we explicitly indicate the species from which experimental evidence was derived, with particular emphasis on findings from rodent models (primarily mice) and dairy ruminants (cattle, goats), while noting areas where evidence from human studies contributes to our understanding.

## 2. Mammary Gland Development

### 2.1. Composition and Structure of the Mammary Gland

The mammary gland, an epidermal-derived organ that evolved from apocrine sweat glands approximately 300 million years ago, is a defining feature of mammals [13]. It is composed of a branching network of ducts and lobuloalveolar structures embedded in a stromal matrix. The basic functional unit—the lobule—contains alveoli lined by secretory luminal epithelial cells and surrounded by contractile myoepithelial cells. These structures connect via a system of ducts that converge toward the nipple [14].

Histologically, the mammary epithelium in mammals consists of two main layers: an inner layer of luminal epithelial cells and an outer layer of basal cells, which include myoepithelial cells and stem/progenitor populations. These layers rest on a basement membrane that provides structural and biochemical signals essential for epithelial organization and function [15,16]. The surrounding stromal microenvironment contains adipocytes, fibroblasts, immune cells, vascular and neural elements, all contributing to gland regulation through reciprocal epithelial–stromal crosstalk [17,18]. These interactions, mediated by paracrine and autocrine signaling, are central to mammary morphogenesis and differentiation.

Notably, there are important anatomical and functional differences across species. In dairy ruminants such as cows and goats, the mammary glands are positioned in the inguinal region, forming a large udder composed of four (cow) or two (goat) separate quarters [19,20]. These glands contain a highly developed lobuloalveolar system capable of producing high daily milk volumes [21,22]. In humans, the thoracic mammary glands consist of a single pair of breasts on the chest, each containing approximately 15–20 primary lactiferous ducts that converge at the nipple [23]. In rodents, such as mice, five pairs of mammary glands extend along the ventral trunk, and development emphasizes terminal end buds (TEBs) and rapid ductal branching [18,24]. Similarly, pigs possess 6–7 pairs of mammary glands distributed along the trunk, each functioning independently to support 10–14 offspring, with modest alveolar development relative to dairy ruminants [25]. Despite these anatomical and functional specializations, the fundamental epithelial bilayer organization and core regulatory pathways governing mammary development remain broadly conserved across mammals.

### 2.2. Developmental Stages of the Mammary Gland

**Figure 1:**
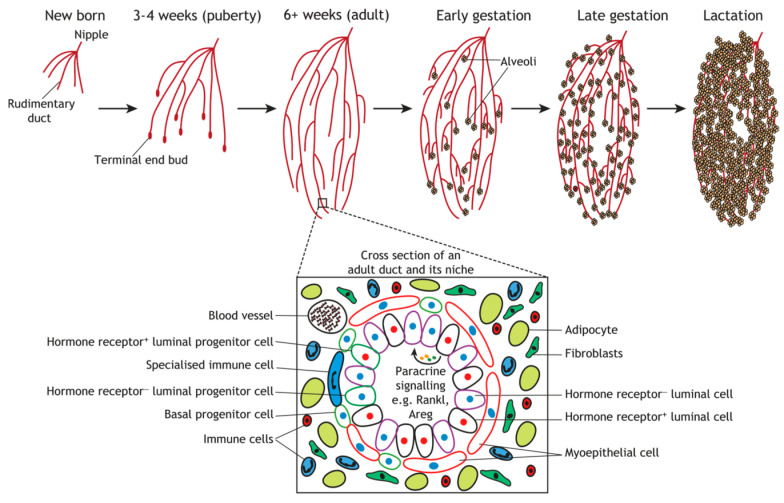
Schematic overview of postnatal mammary gland developmental stages in the mouse. Comprehensive depiction of postnatal mammary gland development illustrating morphological transitions through prepubertal, pubertal, pregnancy, lactation, and involution phases. The main panel demonstrates progressive architectural remodeling of the mammary epithelial ductal network across sequential developmental stages, with emphasis on pregnancy-induced structural modifications. The inset illustrates a cross-sectional view of a mammary duct, depicting the cellular heterogeneity and spatial organization of mammary epithelium. Color-coded cell populations represent distinct progenitor and differentiated luminal and basal epithelial cell lineages (cellular hierarchy detailed in Figure 2). The schematic emphasizes the intimate spatial relationship between the epithelial ductal network and surrounding stromal components, including immune cells, adipocytes, and fibroblasts, which collectively contribute to mammary gland development and tissue homeostasis. The content was Adapted from previous published [50].

The mammary gland represents a dynamic and highly specialized organ that undergoes distinct morphological and functional transitions, initiating during embryonic development and continuing throughout the organism’s lifespan [4,26]. Mammary gland organogenesis exhibits considerable interspecies variation with respect to physiological parameters, anatomical configuration, and endocrine regulation [26]. In most mammalian species, mammogenesis is characterized by five principal developmental stages: embryonic, pubertal, pregnancy-associated, lactational, and involutionary phases [27,28]. Figure 1 illustrates an integrative overview of postnatal mammary gland development, depicting the sequential developmental stages and associated cellular interactions governing these processes.

Each developmental stage is coordinately regulated by endocrine hormones, autocrine and paracrine signaling factors, intracellular signaling cascades, and extracellular matrix (ECM) components [29]. Anatomically, mammary gland positioning exhibits phylogenetic variation: inguinal localization in ruminants, thoracic positioning in primates and elephants, and distributed trunk-associated placement in pigs, rats, and mice [30]. These anatomical differences contribute to distinct developmental dynamics; for example, rodents show rapid ductal elongation driven by terminal end buds, whereas ruminants display slower postnatal parenchymal expansion aligned with their longer gestational and lactational cycles [31,32]. Unlike most organ systems that complete terminal differentiation during embryogenesis, the mammary gland acquires the majority of its specialized structural and functional characteristics postnatally, achieving complete functional maturation exclusively during pregnancy [29,33]. This pattern is conserved across species, although the relative contribution of ductal versus alveolar development differs, with rodents exhibiting extensive ductal branching and cattle undergoing pronounced lobuloalveolar growth [32,34]. Despite these interspecies anatomical differences, ruminants, rodents, and humans demonstrate fundamentally conserved developmental programs [8,35].

The initial phase of mammary gland development occurs during embryogenesis, when the mammary line, placodes, and primitive buds develop under the regulatory control of reciprocal epithelial–mesenchymal interactions, establishing the rudimentary ductal architecture [4,36]. This sequence is broadly shared among mammals, although the timing differs substantially—for example, placode formation appears at embryonic day ~10.5 in mice but around gestational day 30 in cattle [37,38]. This developmental phase proceeds largely independently of hormonal regulation and focuses on establishing the foundational mammary epithelial framework. Initially, bilateral mammary ridges (milk lines) emerge along the ventrolateral body axis from ectodermal tissue. As embryonic development progresses, localized cells within these ridges undergo proliferation and invagination, forming discrete mammary placodes that subsequently develop into mammary buds embedded within the nascent mammary fat pad [36]. These primordial buds give rise to primary solid cords that progressively canalize to form hollow secondary ducts with rudimentary branching patterns and a nascent gland cistern [36]. Concurrently, vascular networks, lymphatic vessels, adipose tissue, and stromal connective tissue are established to provide structural and trophic support for the developing gland [32]. Following parturition, the mammary gland enters a relatively quiescent phase that persists until puberty, when rising systemic hormone levels trigger extensive ductal proliferation and branching morphogenesis [4,18,36]. This quiescent-to-pubertal transition is especially pronounced in rodents, where terminal end buds drive ductal invasion of the fat pad, whereas in ruminants, ductal growth is slower and closely linked to body growth rate.

This postnatal quiescence is disrupted at the onset of puberty, during which the mammary gland undergoes substantial architectural remodeling characterized by rapid ductal elongation, formation of terminal end buds (TEBs), and initiation of lobuloalveolar differentiation [39,40]. TEB-driven ductal growth is a hallmark of rodent mammary development but is absent in ruminants (e.g., cow, sheep, goat), where ductal elongation proceeds without distinct TEB structures [41]. These morphogenetic processes are orchestrated by systemic endocrine hormones, particularly estrogen, growth hormone (GH), and insulin-like growth factor 1 (IGF-1), which function synergistically with local paracrine signaling networks to drive epithelial expansion and establish bidirectional stromal-epithelial communication [42]. The pubertal developmental phase thus establishes the structural architecture requisite for subsequent functional differentiation during pregnancy and lactation [3,43].

Subsequently, pregnancy initiates the most dramatic transformation of mammary tissue, characterized by extensive lobuloalveolar expansion and secretory differentiation, processes primarily mediated by progesterone, prolactin, and placental lactogen hormones [44,45,46]. During this phase, the mammary epithelium undergoes dramatic proliferation and differentiation, with alveolar structures developing the specialized biosynthetic machinery necessary for milk production. Following this preparatory period, throughout lactation, the mammary gland achieves maximal functional capacity, with fully differentiated alveolar epithelial cells actively synthesizing and secreting milk components to provide complete nutrition for offspring [32]. In rats, milk yield reaches its peak during mid-lactation, approximately 7–14 days postpartum, whereas dairy cattle maintain high milk output for several months, illustrating notable species-specific lactation strategies [47,48]. This represents the culmination of mammary gland development, wherein the organ fulfills its primary biological function.

Upon weaning, the mammary gland enters the involution phase, during which alveolar structures undergo controlled regression, and secretory epithelial cells are eliminated through programmed cell death (apoptosis) [49].

Macrophages and other immune cell populations exhibit elevated lysosomal activity to phagocytose apoptotic cells and clear residual milk components, while mammary adipocytes undergo regeneration and differentiation to repopulate stromal compartments previously occupied by secretory epithelium during pregnancy and lactation [49]. Collectively, these sequential developmental stages exemplify the mammary gland as a cyclical and remarkably plastic organ, with growth, differentiation, and regression tightly coordinated by integrated hormonal, genetic, and environmental regulatory networks, thereby ensuring optimal functional capacity for supporting mammalian reproduction.

## 3. Regulatory Mechanisms of Embryonic Mammary Gland Development

**Figure 2:**
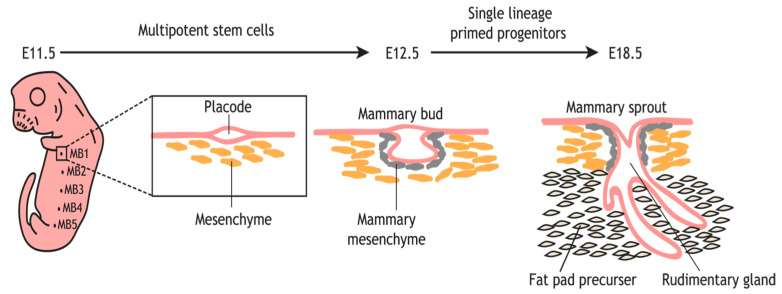
Sequential stages of embryonic mammary gland development. Schematic representation of murine embryonic mammary organogenesis illustrating key morphological transitions and cellular fate specification events. The diagram depicts the initial appearance of mammary placodes at approximately embryonic day (E) 11.5, their progressive development into mammary buds by E12.5, and the subsequent lineage restriction of fetal mammary stem cells (fMaSCs) into distinct basal and luminal epithelial lineages occurring between E12.5 and E18.5. These developmental trajectories and fate specification events have been elucidated through genetic lineage-tracing experiments and single-cell genomic profiling approaches. The figure emphasizes the temporal coordination of morphological transitions with molecular lineage commitment during embryonic mammary development. The content was adapted from previous published study [50].

Embryonic mammary gland development is a highly conserved process initiated by a precise sequence of morphological events, beginning with the formation of an ectodermal thickening known as the mammary band. This structure sequentially evolves into the mammary streak, line, ridge, hillock, and ultimately the mammary bud, which invaginates into the underlying mesenchyme [14,30]. The timing of these events exhibits notable interspecies variation. In murine models, development becomes evident around embryonic day (E) 10.5, whereas in cattle, it commences around gestational day 30 [51], and in pigs, the mammary line appears between days 20–25, with buds forming by days 28–45 [52]. Despite these temporal differences, the core morphological stages and regulatory pathways are largely shared across mammals, establishing a common developmental blueprint. Figure 2 illustrates the sequential stages of embryonic mammary gland development [50].

The molecular regulation of this process is orchestrated by a core set of evolutionarily conserved signaling pathways, primarily operating through reciprocal epithelial–mesenchymal interactions. A key initiating signal is Fibroblast Growth Factor 10 (FGF10), secreted by the mesenchyme, which binds to its receptor FGFR2b on the overlying ectoderm. This FGF10-FGFR2b axis is indispensable for mammary placode formation and subsequent bud morphogenesis, as genetic ablation of either component in mice leads to a severe impairment or complete absence of mammary structures [30,53]. Concurrently, canonical Wnt signaling, mediated by the transcriptional effector Lef1, is critical for the specification of the mammary line and placodes. Lef1 deficiency results in the failure to form multiple mammary placodes, underscoring its fundamental role [54].

As development proceeds, these pathways integrate within a complex regulatory network. The transcription factor Tbx3, for instance, acts upstream by directly regulating the expression of Lef1 and Wnt10b (an early ectodermal marker), and its loss can cause mammary hypoplasia or agenesis [55]. Furthermore, the parathyroid hormone-related protein (PTHrP) signaling axis establishes a crucial paracrine loop; PTHrP from the epithelium signals to its receptor PTHR1 in the mesenchyme, guiding the bud’s extension and initial branching into the mammary fat pad. Studies in mice have demonstrated that disruption of this PTHrP-PTHR1 circuit halts further morphogenesis [56]. Epidermal Growth Factor Receptor (EGFR/ErbB) family signaling also contributes to epithelial cell aggregation and bud differentiation in rodent models, with these signaling pathways appearing functionally conserved across mammalian species [57].

In summary, embryonic mammary morphogenesis is governed by an integrated signaling network where epithelial–mesenchymal feedback is paramount. The mesenchymal-derived FGF10 signal acts as a primary initiator for placode formation. Subsequently, epithelial Wnt and PTHrP signaling engage in a cross-talk with the mesenchyme to consolidate bud identity and direct its invasive growth into the underlying fat pad. Transcription factors like Tbx3 and Lef1 sit at the nexus of these pathways, coordinating the genetic program that translates extracellular signals into morphological change. It is noteworthy that these same pathways—particularly FGF, Wnt, and TGF-β family members—are repurposed in postnatal life to regulate ductal branching and alveolar expansion, demonstrating a remarkable conservation of the molecular toolkit throughout the gland’s development (Table 1).

## 4. Regulatory Mechanisms in Pubertal Mammary Gland Development

Puberty marks a critical phase in mammary gland development, initiated by the activation of the hypothalamic–pituitary–gonadal axis and the consequent surge in systemic hormones. This period is defined by the transformation of a simple, rudimentary ductal network into an elaborate, branched architecture, a process essential for preparing the gland for its future functional role in pregnancy and lactation [39,42]. During early postnatal life, the mammary gland in calves grows isometrically with the rest of the body. Between 2 and 3 months of age, this transitions to an allometric growth phase, marked by rapid ductal extension and fat pad expansion, primarily stimulated by estrogen (E2), growth hormone (GH), and insulin-like growth factor-1 (IGF-1) [5]. Around 9 months, the duct system develops into a complex branching network. The ends of the ducts form terminal ductal units (TDUs), which drive further growth and branching until they reach the boundaries of the fat pad [53,73,74]. This process is supported by developing vascular and lymphatic networks [75]. Locally produced transforming growth factor-β (TGF-β) later inhibits further branching, shaping the final ductal tree [5,56]. Notably, high-energy nutrition during allometric growth can increase stromal fat and reduce parenchymal tissue, impairing development [76,77].

This complex remodeling is orchestrated by a tightly coordinated interplay of systemic hormones and local signaling cascades that regulate epithelial cell proliferation, differentiation, and spatial organization [40,78]. Estrogen, acting primarily through its receptor ERα, is the principal mitogen for ductal elongation, as evidenced by the severely stunted ductal networks in ERα-deficient mice [79,80,81,82]. A key mechanism of estrogen action is the induction of the epidermal growth factor receptor (EGFR) ligand, amphiregulin (AREG). Estrogen-stimulated ERα+ cells secrete AREG, which in a paracrine manner activates EGFR on neighboring cells to drive proliferation and TEB formation [83,84]. Parallel mechanisms have been identified in humans, while in ruminants estrogen drives ductal elongation through AREG-mediated pathways despite the absence of classical TEB structures [85]. The NOTCH pathway, acting through its four receptors, controls cell communication and development and is a key regulator of adolescent mammary growth in humans, mice, and cattle [86]. Thus, although the morphologies differ, estrogen-AREG–EGFR signaling constitutes a conserved regulatory axis across species.

The GH–IGF-1 axis plays a central role in coordinating epithelial proliferation and ductal branching during puberty. GH stimulates stromal production of IGF-1, which acts locally to promote proliferation and support ductal expansion [87,88,89,90]. In ruminants, this pathway is especially important: exogenous GH administration enhances mammary development in heifers and supports allometric parenchymal growth [90,91,92]. Progesterone, signaling through its receptor (PR), is particularly important for mediating lateral branching, and the balanced expression of its PR-A and PR-B isoforms is crucial for normal ductal patterning [93,94,95,96]. Studies in bovine demonstrate that estrogen and progesterone act synergistically to enhance epithelial proliferation and generate a more complex ductal network than either hormone alone [97].

The systemic hormonal signals are intricately relayed and refined through stromal-epithelial crosstalk. A pivotal player in the stroma is the transcription factor GLI2, which regulates the expression of estrogen and GH receptors in stromal cells and promotes the production of paracrine factors like WNT ligands and IGF-1. These stromal-derived signals are essential for driving the proliferation of mammary epithelial cells and proper TEB function [89,98]. The canonical WNT/β-catenin pathway acts as a crucial intracellular signaling hub within TEBs, promoting the expression of cell cycle genes and facilitating ductal invasion [99]. Similar WNT-dependent mechanisms operate in cattle and humans, although functional characterization remains most extensive in rodent models [100]. Additional guidance molecules, including netrin-1, slit2, and reelin, along with matrix metalloproteinases (MMPs), coordinate ductal invasion and ECM remodeling across species [4,86].

The transcriptional output of these coordinated signals is a highly dynamic gene expression profile. Upregulated genes during puberty include those involved in the cell cycle, serotonin signaling (e.g., receptors *5-HTR1A* and *5-HTR1B*), and targets of the Notch pathway (e.g., *HEY1*, *HEY2*) [86,101]. Serotonin has been shown to synergize with estrogen to influence mammary development [101], while IGF1 signaling activates PI3K/AKT and MAPK pathways to promote cell proliferation and survival, often in conjunction with the Notch pathway [102].

**Figure 3:**
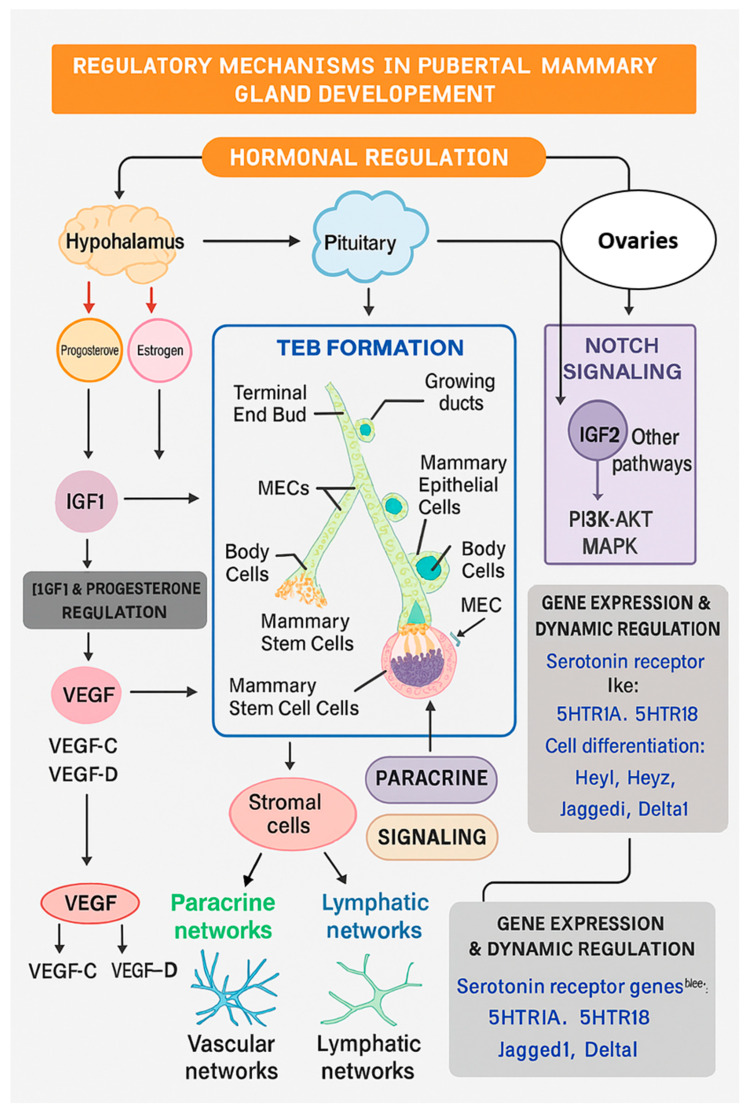
Integrated regulatory networks governing pubertal mammary gland development. Schematic representation illustrating the multifaceted regulatory mechanisms orchestrating pubertal mammary morphogenesis. The diagram depicts the hierarchical organization of hormonal regulation, initiated by hypothalamic–pituitary–gonadal axis activation, which stimulates ovarian secretion of progesterone and estrogen. These hormones, in conjunction with pituitary-derived factors, promote terminal end bud (TEB) formation and ductal elongation. The central panel illustrates TEB architecture, showing the spatial organization of cap cells, body cells, mammary epithelial cells (MECs), and mammary stem cells within the proliferative structure. Progesterone and estrogen indirectly stimulate insulin-like growth factor 1 (IGF1) production, which synergistically regulates mammary development. The right panel depicts Notch signaling pathway activation in ovarian tissues, leading to IGF2 production and downstream activation of PI3K-AKT and MAPK signaling cascades. Paracrine signaling mechanisms are illustrated through stromal cell-mediated communication, whereby stromal cells respond to systemic hormones and secrete paracrine factors that promote vascular and lymphatic network development through VEGF-C and VEGF-D signaling. The lower panels highlight key gene expression programs and dynamic transcriptional regulation during pubertal development, including upregulation of serotonin receptor genes (*5HTR1A*, *5HTR1B*) and Notch signaling components (Hey1, Hey2, Jagged1, Delta1). This integrative framework emphasizes the coordinated interaction between endocrine hormones, paracrine growth factors, and intracellular signaling cascades that collectively drive pubertal mammary gland morphogenesis.

In summary, pubertal mammary gland development is governed by a hierarchical regulatory network. Systemic estrogen, relayed via stromal-epithelial loops and paracrine effectors like AREG/EGFR, initiates ductal elongation and TEB formation. GH (via IGF-1) and progesterone synergize with estrogen to promote branching and proliferation. These systemic cues are spatially coordinated by local stromal signals (WNT, IGF-1), cell–cell communication pathways (Notch), and ECM remodeling, while being ultimately bounded by inhibitory signals like TGF-β. The concerted action of these endocrine and paracrine networks constructs the mature ductal tree, establishing the foundational architecture necessary for future functional differentiation. Figure 3 provides a visual integration of these core regulatory mechanisms and their interactions.

## 5. Regulatory Mechanisms of Mammary Gland Development During Pregnancy

During pregnancy, mammary development shifts from pubertal ductal expansion to lobulo-alveolar growth and functional maturation. In early gestation, mammary epithelial cells (MECs) proliferate extensively to expand alveolar progenitor populations, whereas mid-to-late gestation is dominated by secretory differentiation and establishment of the biosynthetic machinery required for lactation [103]. During pregnancy, human breast development shifts from ductal extension to functional maturation. Early on, matrix metalloproteinases mediate rapid mammary epithelial cell proliferation and epithelial–mesenchymal transition, forming initial acinar structures [104,105,106]. By the second trimester, proliferation slows and differentiation begins, with acini surrounded by capillary networks for nutritional support [107]. In late pregnancy, progesterone and prolactin stimulate gland maturation, marked by apical villous differentiation in epithelial cells, indicating structural and functional readiness for lactation [50].

Pregnancy-associated morphogenesis requires active extracellular matrix remodeling. Matrix metalloproteinases (e.g., MMP-3) and related proteases facilitate ductal side-branching, epithelial expansion, and formation of nascent alveoli by modifying the stromal microenvironment [104,105,106,108,109,110,111]. Although mechanistic evidence for EMT-linked remodeling is strongest in rodents, bovine studies support a role for MMP-mediated structural remodeling during alveolar development [108,109,110,111]. In the early stages of pregnancy, mammary epithelial cells (MEC) proliferate rapidly, mediated by matrix metalloproteinases such as MMP-3, and form alveolar structures through the epithelial–mesenchymal transition (EMT). This transition involves the down-regulation of e-cadherin and β-catenin, alongside the up-regulation of vimentin, which is essential for branched morphogenesis [108,109,110,111]. As gestation advances, MEC proliferation slows and differentiation increases, accompanied by enhanced vascularization around developing alveoli to support nutrient delivery [107,112]. These structural changes are controlled by coordinated endocrine signals. Estrogen and progesterone drive ductal and alveolar expansion throughout gestation [2,113,114,115]. Progesterone also suppresses premature expression of milk protein genes (e.g., β-casein), preventing early lactogenesis before parturition [116,117]. Species differences exist in hormonal sensitivity; for instance, PR expression remains relatively low in early–mid gestation in yaks and Tibetan pigs, indicating delayed progesterone responsiveness compared with dairy cattle.

The endocrine milieu is dominated by the concerted actions of progesterone (P), estrogen (E2), and prolactin (PRL). Circulating prolactin levels, for instance, rise progressively throughout pregnancy, reaching up to 10-fold higher concentrations by the third trimester in humans, a surge that correlates directly with increasing mammary gland activity [118,119]. Prolactin is the principal lactogenic hormone, driving alveolar development, activating the transcription of milk protein genes, and later regulating tight junction closure and postpartum milk synthesis in response to suckling [119,120,121]. In rodent models, prolactin also exerts central effects to modulate maternal behavior and food intake, thereby indirectly supporting lactation [122,123,124]. The essential roles of these hormones are underscored by genetic studies; the knockout of their respective receptors severely disrupts normal mammary development [125]. Findings in yaks and Tibetan pigs, where progesterone receptor (PR) expression remains low during early to mid-pregnancy, highlight species-specific variations in the temporal dynamics of hormonal sensitivity [126]. The synergistic action of E2, P, and PRL is crucial for the coordinated ductal and alveolar expansion observed during this period [127].

These systemic hormonal signals are transduced into cellular responses through key intracellular pathways. In rodents and ruminants, the JAK2/STAT5 axis is a master regulator for alveolar formation and the transcription of milk protein genes, with STAT5 phosphorylation and nuclear translocation being pivotal for initiating and maintaining lactation [128,129,130]. Concurrently, the PI3K/Akt pathway is integral for regulating MEC survival, growth, and proliferation throughout pregnancy [131,132]. Research in bovine models, such as that by Meng et al., confirms the critical importance of these pathways, particularly after mid-pregnancy, for mammary development and the transition to lactation [133].

In summary, mammary gland development during pregnancy is a temporally coordinated process governed by a hierarchical regulatory network. The systemic endocrine environment, defined by rising levels of progesterone, estrogen, and prolactin, sets the overarching program. These hormones activate core signaling cascades, primarily JAK2/STAT5 and PI3K/Akt, to drive the biphasic cellular response of proliferation followed by differentiation. This hormonal and signaling framework subsequently directs stage-specific transcriptional and metabolic programs—from early metabolic preparation and mid-gestation proliferative expansion to late-gestation immune preparation and functional maturation—ensuring the gland is fully equipped for the imminent demands of lactation.

## 6. Mammary Gland Development During Lactation

Lactation represents the final and most functionally demanding stage of mammary gland development, where the gland achieves its peak functional state through profound physiological and biochemical reprogramming to sustain milk production [43]. Morphologically, the mammary parenchyma undergoes remarkable expansion, with the adipose-rich stroma being largely replaced by numerous, milk-distended alveoli that fill the luminal spaces [43,134]. This secretory architecture is supported by critical ultrastructural adaptations in alveolar epithelial cells, including enhanced mitochondrial density for energy metabolism, extensive development of the rough endoplasmic reticulum for protein synthesis, and heightened Golgi apparatus activity for post-translational modification and secretion of milk components [135]. These morphological changes are conserved across mammals, although the degree of lobuloalveolar expansion is considerably greater in dairy ruminants than in rodents or humans due to their substantially higher milk yields.

The molecular landscape of the lactating gland reflects a highly coordinated anabolic state, enabling simultaneous synthesis of milk proteins, lipids, and lactose. Transcriptomic studies in Holstein cows report 122 differentially expressed genes during lactation relative to virgin and dry periods, with 79 genes significantly upregulated [136]. This pattern is evolutionarily conserved, as similar transcriptional shifts toward a secretory phenotype are observed in murine models [137,138]. The upregulated genes form a coordinated network that drives the synchronized production of milk’s major components. Milk fat synthesis is enabled through the concerted upregulation of genes governing fatty acid uptake (*LPL*), intracellular transport (*FABP3*), and de novo synthesis (*ACACA*), while fatty acid oxidation pathways are concurrently suppressed [139]. This lipogenic program is complemented by sustained high expression of casein genes like *CSN2* (β-casein), which is coregulated with genes involved in lactose synthesis to ensure the production of milk’s primary nutritional components [140]. In ruminants, the magnitude of these transcriptional changes is larger due to prolonged lactation and greater metabolic load compared with rodents and humans.

Lactation is accompanied by profound metabolic reprogramming, driven by hormonal cues and sustained milk removal [141]. Proteomic studies in bovine mammary tissue have identified 21 proteins upregulated during lactation, many associated with metabolic pathways and nutrient transport [142]. Transcriptomic datasets confirm that the onset of lactation is characterized by increased expression of genes for carbohydrate, amino acid, and lipid metabolism, whereas cell-cycle-associated genes become downregulated as cells shift from proliferation to secretion [143]. System-wide adaptations also occur: the liver upregulates genes involved in basal metabolism, while the mammary gland increases the expression of nutrient transporters and signaling pathways such as MAPK, WNT, and JAK–STAT to support milk production [144]. Rodent MECs similarly exhibit elevated metabolic activity during lactation under the control of insulin-dependent pathways, although their metabolic demands are proportionally lower due to shorter lactation duration [145]. In dairy cattle, intense anabolic activity requires substantial blood flow, with approximately 400 L of blood passing through the udder per liter of milk synthesized [35].

The longevity and productivity of lactation in cattle—often exceeding 300 days—depend on precise molecular control [146]. Milk yield declines following the natural reduction in estrogen after peak lactation [27]. As lactation advances to its late stage, the gland initiates a preparatory transition toward involution. The gene expression profile undergoes a strategic shift from biosynthetic dominance to remodeling priority. This transition is marked by the upregulation of pro-apoptotic mediators including CASP8 and BAX, coupled with downregulation of the anti-apoptotic gene BCL2, initiating the first phase of epithelial regression [147]. Concurrently, the expression of key transcriptional regulators of lipid synthesis, PPARG1 and SREBF1, declines significantly, leading to reduced milk fat synthesis capacity [148]. This metabolic downregulation is further promoted by elevated expression of inflammatory cytokines such as IL6 and TNF-α, which activate signaling cascades that will ultimately culminate in full-scale involution [147]. These coordinated molecular changes in late lactation effectively bridge the functional transition from active milk synthesis to the regenerative phase of the dry period, ensuring a controlled progression through the mammary developmental cycle.

## 7. Mammary Gland Development During the Dry Period

The dry period represents a critical non-lactating phase in the lactation cycle, typically spanning 40 to 60 days in dairy cattle, during which the mammary gland undergoes comprehensive restructuring to regenerate its functional capacity and prepare for the subsequent lactation [149,150]. This stage is characterized by two distinct physiological processes: the extensive involution of the secretory epithelium followed by a phase of cellular renewal and regeneration, both of which are essential for optimizing milk yield and quality in the next lactation cycle [151].

The degeneration of bovine mammary glands occurs in two distinct stages. In the initiation stage, It initiates with the cessation of milking and is primarily characterized by the apoptosis of mammary epithelial cells (MECs) and the disruption of tight junctions (TJs). A key regulatory signal in this phase is the activation of STAT3. Within 72 h after milking stops, signaling molecules such as leukemia inhibitory factor (LIF) activate STAT3 [152]. Activated STAT3, on one hand, upregulates the expression of suppressor of cytokine signaling 3 (SOCS3). SOCS3, by inhibiting the activity of STAT5, blocks prolactin-induced transcription of milk protein genes, thereby shutting down the lactation program [152]. On the other hand, STAT3 also regulates the uptake of milk fat globules and increases lysosomal membrane permeabilization, ultimately leading to epithelial cell death [153,154]. Concurrently, the expression of insulin-like growth factor-binding protein 5 (IGFBP-5) increases. By binding to and inhibiting the survival signal molecule IGF-1, IGFBP-5 further promotes the apoptosis of MECs [155]. It is noteworthy that, compared to rodents, bovine mammary gland involution occurs at a slower rate, partly due to lower expression levels of IGFBP-5 and stronger activity of the IGF1-AKT survival signaling pathway [152,156]. This phase also involves the degradation of β-casein (CSN2) by plasmin. The resulting peptides can block potassium channels on the apical membrane of MECs, thereby inhibiting milk secretion [157]. As the tissue structure is not yet completely destroyed, this phase is reversible upon suckling stimulation [152].

Upon entering the second stage of the degeneration process, this phase is characterized by extensive remodeling of the extracellular matrix (ECM) and the large-scale clearance of epithelial cells. Its core involves the systemic activation of various proteolytic enzymes, including matrix metalloproteinases (MMPs), plasminogen activators, cathepsin B, and serine proteases [158,159]. These enzymes act in concert to degrade basement membrane and ECM components, such as laminin and collagen. The disruption of the ECM severs the anchorage-dependent survival signals for MECs, accelerates the apoptosis process, and leads to the complete collapse of the lobuloalveolar structures [108,160]. Furthermore, transforming growth factor-β3 (TGF-β3) is induced during this stage. It further drives the involution process by promoting the programmed cell death of MECs and suppressing potential lactogenic activity [161]. Another important regulator is Mammary Gland Protein-40 (MGP-40), a chitinase-like protein highly expressed during involution. Although it lacks chitinase activity, it is believed to play a role in mammary tissue remodeling and progenitor cell protection [162]. Ultimately, the apoptotic MECs are cleared by phagocytosis, carried out by macrophages and neighboring viable epithelial cells. Adipocytes subsequently repopulate the tissue space, restoring the mammary gland to a quiescent, pre-pregnancy-like state in preparation for the next lactation cycle [158,163].

The involution process is initiated by the dramatic withdrawal of lactogenic hormones. The decline in circulating prolactin, estrogen, and progesterone following the cessation of milking removes the primary trophic signals for milk synthesis, while reduced oxytocin levels contribute to teat canal closure and the complete cessation of milk removal [164,165]. This hormonal shift triggers a cascade of molecular events, beginning with milk stasis within the alveolar lumen. The accumulated milk creates physical pressure that synergizes with intracellular signaling pathways to initiate programmed cell death [166]. A key early mediator is interleukin-6 (IL-6), whose expression rises significantly during involution after remaining suppressed throughout lactation. IL-6 activates both the STAT3 and p44/42 MAPK signaling cascades, with studies in IL-6-null mice demonstrating a delayed involution process, confirming its pivotal role in initiating epithelial regression [167].

The molecular execution of involution involves multiple coordinated pathways. Transforming growth factor-beta (TGF-β) emerges as a major pro-apoptotic factor that not only promotes epithelial cell apoptosis but also facilitates the clearance of dying cells and initiates extracellular matrix (ECM) remodeling—processes that may influence the gland’s susceptibility to tumorigenesis during this transitional window [168]. Concurrently, the glandular stroma undergoes profound reconstruction, with fibroblasts becoming increasingly active in producing new ECM components while adipocytes progressively repopulate the stromal compartment to restore the mammary fat pad architecture [169].

Environmental factors significantly impact the efficiency of these regenerative processes. Research from the Laporta group has demonstrated that heat stress during the dry period substantially compromises mammary regeneration through multiple interconnected mechanisms. Exposure to high temperature–humidity conditions not only reduces mammary epithelial proliferation but also disrupts the expression of genes governing cell cycle regulation, protein folding, and immune function [170,171]. At a mechanistic level, heat stress impairs critical cellular processes including autophagy and apoptosis—both essential for proper mammary epithelial cell turnover and renewal—through downregulation of key autophagy-related genes such as *ATG3* and *ATG5* [170,172,173]. These cellular alterations are accompanied by systemic endocrine and metabolic changes, including altered prolactin signaling and reduced IGF-1 bioavailability, which collectively impair epithelial cell survival and proliferative capacity [171]. Importantly, their research has revealed that the effects of heat stress extend beyond the dam, as calves born to heat-stressed dams exhibit altered metabolic and immune profiles, indicating transgenerational programming of mammary development [174].

In contrast to the detrimental effects of heat stress, photoperiod management represents a positive environmental strategy to enhance mammary regeneration. Short-day photoperiods during the dry period enhance prolactin receptor expression and downstream signaling, which promotes mammary epithelial cell proliferation through upregulation of key cell cycle regulators including *Cyclin D1* and *CDK4*, thereby improving parenchymal regeneration prior to calving [175].

The transcriptional landscape during the dry period reflects this shift from a secretory to a regenerative state. Global gene expression analyses reveal systematic downregulation of genes involved in milk synthesis, while genes associated with apoptosis (*BAX*, *CASP8*), tissue remodeling, and immune responses are markedly upregulated [151,176,177]. This genetic reprogramming is further evidenced by the abundant expression of lysozymes, hydrolases, and cathepsins by day 7 after weaning, which function to degrade apoptotic cell debris and residual milk components [178]. Simultaneously, genes encoding ECM proteins, cell adhesion molecules, and basement membrane components are upregulated, facilitating the reconstruction of the gland’s structural framework in preparation for the next lactation cycle [179].

The duration of the dry period represents a critical determinant of regenerative success. Shortened dry periods (≤40 days) consistently impair mammary cell renewal and subsequent milk production due to insufficient time for completion of the biphasic involution-regeneration process. Mechanistically, this compressed timeline disrupts the normal progression of apoptosis, autophagy, and stromal remodeling, ultimately limiting the population of regenerated secretory epithelial cells [180,181]. While some studies suggest that extending the dry period to approximately 13 weeks may not significantly affect milk yield in the subsequent cycle, prolonged dry periods frequently lead to excessive body condition with heightened risks of peripartum metabolic disorders and may effectively reduce productive lifespan by shortening the subsequent lactation period, thereby incurring substantial milk yield losses [182,183]. This underscores the importance of optimal dry period length for balancing mammary gland renewal and productivity across lactation cycles.

Nutritional management during the prepubertal period has a profound impact on mammary parenchymal development and future milk yield [184,185]. Classic studies in heifers demonstrated that an excessively high plane of nutrition accelerates body growth but may reduce the relative growth of mammary parenchyma and increase fat deposition in the gland, potentially compromising first-lactation yield [186]. One recent study founded that preweaning nutrient intake significantly influenced mammary gland development in dairy heifer calves, with those fed an enhanced diet showing markedly greater mammary parenchymal growth and total stem cell population, indicating that allometric mammary growth can be initiated before weaning [187]. Similarly, Meyer et al. (2006) quantitatively analyzed parenchymal and fat pad mass and composition in Holstein heifers under differing nutrient regimens, confirming that the mammary fat pad responds strongly to increased nutrient intake while parenchymal tissue remains more conserved in early stages [188,189]. During late gestation, maternal nutrition also influences prepartum mammary epithelial proliferation and colostrum quality, with potential carry-over effects on neonatal performance and subsequent lactations [184]. Balanced nutrition during early growth and gestation is crucial to promote healthy mammary development and sustain high milk yield potential.

## 8. Conclusions

Mammary gland development represents a highly dynamic process orchestrated by complex interactions among endocrine hormones, growth factors, and conserved signaling pathways, including Wnt, Notch, TGF-β, PI3K/Akt, and JAK2/STAT5. Each developmental stage—from embryonic specification through puberty, pregnancy, lactation, and involution—is regulated by distinct molecular programs that ensure appropriate functional specialization. The dry period plays a critical yet often underappreciated role in mammary regeneration and preparation for subsequent lactation cycles. Environmental factors, particularly heat stress and photoperiod, significantly influence mammary development and lactational performance, emphasizing the importance of optimized management strategies in dairy production. Future research should focus on elucidating epigenetic mechanisms, non-coding RNA regulation, and extracellular vesicle-mediated signaling to advance both fundamental understanding and practical applications in mammary biology.

Looking forward, several emerging research directions promise to deepen our understanding of mammary gland biology. The application of single-cell multi-omics technologies will be crucial for delineating cellular heterogeneity and lineage commitment throughout development, particularly in identifying novel progenitor subpopulations and their regulatory networks. There is growing recognition that non-coding RNA networks, including microRNAs and long non-coding RNAs, serve as key post-transcriptional regulators of mammary development, yet their specific roles across different developmental transitions remain largely unexplored. Extracellular vesicle-mediated signaling represents another frontier, with emerging evidence suggesting that milk-derived exosomes and their cargo may coordinate both local tissue remodeling and systemic physiological adaptations. Furthermore, the field would benefit from increased investigation into transgenerational epigenetic regulation, particularly how environmental exposures during critical developmental windows (e.g., maternal heat stress during the dry period) program offspring mammary development through DNA methylation and histone modifications. Integrating these molecular insights with physiological outcomes through systems biology approaches will be essential for developing novel strategies to enhance mammary health and productivity in dairy production systems. Future research bridging fundamental mammary biology with practical agricultural applications will ultimately support the development of precision management strategies that optimize mammary development and lactational performance across diverse production environments.

## Figures and Tables

**Table 1:** Signaling pathways associated with mammary gland development/remodeling.

Periods of Possible Impact	Signaling Pathway	Gene/Transcription Factor	References
Embryonic, pubertal, and pregnancy stages	Wnt	*Wnt3*, *Wnt6*, *Wnt10b*, *Wnt4*, *LEF1*, *LEF*, *β-catenin*	[58,59]
Embryonic, pubertal, and pregnancy stages	BMP	*BMP2*, *BMP4*	[60,61]
Embryonic, pubertal, and pregnancy stages	PTHLH	*PTHrP*, *PTH1R*	[56]
Embryonic, pubertal, and pregnancy stages	Hedgehog	*SHH*, *PTCH1*, *GLI3*	[62,63]
Embryonic, pubertal, and pregnancy stages	FGF	*FGF10*, *FGFR2b*	[53,64]
Embryonic, pubertal, and pregnancy stages	Notch	*NOTCH2*, *NOTCH4*	[65]
Pregnancy, lactation, and involution stages	JAK-STAT	*STAT5a*, *STAT5b*, *STAT6*, *STAT3*, *LIF*	[4,65]
Pregnancy, lactation, and involution stages	RANKL-RANK	*RANKL*	[66]
Embryonic, pubertal, pregnancy, lactation, and involution stages	PI3K-Akt	*PI3K*, *Akt1*, *mTOR*, *PIP2*, *PIP3*, *PTEN*, *Cyclin D1*	[67]
Pubertal, pregnancy, lactation, and involution stages	NF-κB	*NF-κB*, *IKK*, *RelA*, *p65*, *p50*	[68,69]
Embryonic, pubertal, pregnancy, and involution stages	TGF-β	*TGF-β1*, *TGF-βR*, *SMAD2*, *SMAD3*, *SMAD4*	[69]
Pubertal, pregnancy, and lactation stages	MAPK	*RAS*, *RAF*, *MEK*, *ERK*	[70,71]
Pubertal, pregnancy, lactation, and involution stages	Autophagy	*ATG5*, *Beclin1*, *LC3*	[72]

## Data Availability

No new data were created or analyzed in this study. Data sharing is not applicable.
